# Human Sperm Capacitation Involves the Regulation of the Tyr-Phosphorylation Level of the Anion Exchanger 1 (AE1)

**DOI:** 10.3390/ijms21114063

**Published:** 2020-06-05

**Authors:** Gabriella Donà, Elena Tibaldi, Alessandra Andrisani, Guido Ambrosini, Chiara Sabbadin, Mario Angelo Pagano, Anna Maria Brunati, Decio Armanini, Eugenio Ragazzi, Luciana Bordin

**Affiliations:** 1 Department of Molecular Medicine-Biological Chemistry, University of Padua, 35131 Padua, Italy; gabriella.dona@unipd.it (G.D.); elena.tibaldi@unipd.it (E.T.); mario.pagano@unipd.it (M.A.P.); annamaria.brunati@unipd.it (A.M.B.); 2 Department of Women’s and Children’s Health, University of Padua, 35131 Padua, Italy; alessandra.andrisani@unipd.it (A.A.); guido.ambrosini@unipd.it (G.A.); 3 Department of Medicine–Endocrinology, University of Padua, 35131 Padua, Italy; chiarasabbadin.85@gmail.com (C.S.); decio.armanini@unipd.it (D.A.); 4 Department of Pharmaceutical and Pharmacological Sciences, University of Padua, 35131 Padua, Italy; eugenio.ragazzi@unipd.it

**Keywords:** sperm, SLC4A1, AE1, Tyr-phosphorylation, capacitation, acrosome reaction, Lyn, Syk

## Abstract

Bicarbonate uptake is one of the early steps of capacitation, but the identification of proteins regulating anion fluxes remains elusive. The aim of this study is to investigate the role of sperm solute carrier 4 (SLC4) A1 (spAE1) in the capacitation process. The expression, location, and tyrosine-phosphorylation (Tyr-P) level of spAE1 were assessed. Thereby, it was found that 4,4′-Diisothiocyano-2,2′-stilbenedisulfonic acid (DIDS), an SLC4 family channel blocker, inhibited capacitation in a dose-dependent manner by decreasing acrosome reaction (ARC% 24.5 ± 3.3 vs. 64.9 ± 4.3, *p* < 0.05) and increasing the percentage of not viable cells (NVC%), comparable to the inhibition by I-172, a cystic fibrosis transmembrane conductance regulator (CFTR) blocker (AR% 30.5 ± 4.4 and NVC% 18.6 ± 2.2). When used in combination, a synergistic inhibitory effect was observed with a remarkable increase of the percentage of NVC (45.3 ± 4.1, *p* < 0.001). spAE1 was identified in sperm membrane as a substrate for Tyr-protein kinases Lyn and Syk, which were identified as both soluble and membrane-bound pools. spAE1-Tyr-P level increased in the apical region of sperm under capacitating conditions and was negatively affected by I-172 or DIDS, and, to a far greater extent, by a combination of both. In conclusion, we demonstrated that spAE1 is expressed in sperm membranes and it is phosphorylated by Syk, but above all by Lyn on Tyr359, which are involved in sperm viability and capacitation.

## 1. Introduction

Capacitation, a critical step in the maturation of sperm is characterized by a series of biochemical and physiological changes both in the head (preparation for the acrosome reaction, AR) and in the tail (hyperactivation) of sperm to gain the ability to fertilize oocyte.

Although a few controversies over as yet unknown mechanisms remain to be addressed, the increase in intracellular bicarbonate (HCO_3_^−^) is widely accepted as one of the first and essential events taking place during capacitation [1]. 

HCO_3_^−^ uptake induces the activation of the atypical soluble adenylyl cyclase (ADCY10) which, in turn, leads to cAMP synthesis and the activation of protein kinase A (PKA) [2]. This signaling pathway initiates downstream events such as plasma membrane hyperpolarization and cytoplasm alkalinization [1,3]. The membrane HCO_3_^−^ transporters^-^ are traditionally classed into two main protein families, solute carrier 26 (SLC26), acting as anion exchangers, and solute carrier 4 (SLC4), acting as either exchangers or cotransporters [4,5], to which the cystic fibrosis transmembrane conductance regulator (CFTR) can be added, this latter functioning as an ATP-gated anion channel [6]. Importantly, CFTR, SLC26 with its members SLC26A3, SLC26A6, and SLC26A8, as well as SLC4, represented by the member SLC4A1, have been demonstrated to be expressed also in mature spermatozoa [1,7,8,9,10,11].

CFTR is activated by PKA-mediated phosphorylation [12,13]. In this regard, an initial HCO_3_^−^ entry seems to be indispensable for the cAMP increase, leading to PKA activation which, in turn, is thought to further sustain HCO_3_^−^ entry by phosphorylating CFTR. To date, the potential player responsible for this initial HCO_3_ transport is yet to be identified.

Previous studies showed that CFTR inhibition resulted in low PKA activity and partial inhibition of cAMP/PKA-related downstream events such as the increase in tyrosine phosphorylation (Tyr-P), hyperactivated motility, and AR [13]. The lack of total inactivation of the PKA-CFTR related pathway in the presence of CFTR blocker is consistent with the occurrence of an additional HCO_3_^−^ transporter that may cooperate in the bicarbonate balance inside the cell.

The SLC4 family is the major group of membrane HCO_3_^−^ transporters, comprising ten protein members [4]. Among them, SLC4A1/AE1 has been shown to mediate 1:1 electroneutral exchange of many monovalent anions under experimental conditions, but Cl^−^ and HCO_3_^−^ are its major physiological substrates. AE1, also known as band 3, is the most expressed anion exchanger in several different tissues, such as intestine, kidney, heart, and others [4]. In human erythrocytes, AE1 (eAE1) represents the most expressed protein in the plasma membrane, accounting for about one quarter of the cell total protein content. In the male reproductive tract, AE1 is expressed in the epididymal tissues [14], and has also been shown to be present in mature sperm [8], but compelling evidence for AE1 actively taking part in the regulation of any of the physiological pathways of human sperm remains elusive.

eAE1 is located in the plasma membrane as a dimer or tetramer, composed of 14 transmembrane-spanning segments and the N- and C-termini facing the inner side of the plasma membrane. Asn642 is the N-glycosylation site on the outer side of the cell surface, whereas the cytosolic domains of eAE1 contain binding sites for a number of proteins and enzymes, including glycolytic enzymes [15], hemoglobin [16], and cytoskeletal proteins. The interactions between eAE1 and other protein partners are regulated by the phosphorylation state of four residues, namely Tyr8, Tyr21, Tyr359, and Tyr904, which are targeted by the tyrosine kinases Syk (Tyr8, Tyr21 and Tyr904) [17] and Lyn (Tyr359) [18]. 

Human capacitation involves several simultaneous and self-regulating processes: as bicarbonate entry triggers PKA activity and the downstream pathway leading to an increase in Tyr-P [19], other events seem to contribute to the Tyr-P level regulation. First, endogenous production of reactive oxygen species (ROS) increases Tyr-P level by inhibiting protein phosphatase activity [20,21]. In addition, ROS-related oxidation, along with cholesterol efflux, induces plasma membrane rearrangement and the displacement of membrane microdomains (rafts) to the sperm head [22]. Rafts harbor several proteins, including enzymes that are placed close to relevant substrates, which is the case of the tyrosine kinase Lyn, an Src family kinase which has been described to shift to the sperm head during capacitation [23].

After capacitation, sperm cells can undergo the AR, which is the crucial process for fertilization to occur. AR consists in the release of digestive enzymes that are contained in the acrosome, a single, large, dense-core secretory granule overlying the nucleus, allowing them to come into close contact and fuse with an oocyte. Importantly, all the events underlying the complex signaling network that regulates the AR are ultimately mediated by elevation of intra-cellular calcium levels. The calcium ionophore (A23187)-mediated calcium increase activates phospholipase C (PLC), generating inositol 1,4,5-trisphosphate (IP3) and diacylglycerol (DAG). IP3 and DAG THEN act as second messengers to activate IP3 receptor (IP3R) and protein kinase C, both involved in the AR regulation [24]. However, the identification of proteins modulating and undergoing phosphorylation processes is still incomplete. 

The aim of this study was to assess whether sperm SLC4A1 (spAE1) is directly involved in the capacitation process and contributes to the rearrangement of the sperm membranes and to the AR. Moreover, we explored the mechanisms underlying the Tyr-P process in sperm cells in light of previous evidence highlighting the phosphorylation of AE1 in somatic cells, erythrocytes in particular (eAE1), which was shown to result from the sequential activation of the tyrosine kinases Syk and Lyn [17].

## 2. Results 

### 2.1. Effect of the Inhibition of CFTR and SLC4 Family Channels on Sperm AR and Viability

#### 2.1.1. Effect of I-172 and DIDS on Acrosome Reaction (AR) and Sperm Viability

To study the involvement of SLC4 family channels in sperm capacitation and functioning, we compared the effects of 4,4′-diisothiocyano-2,2′-stilbenedisulfonic acid (DIDS), a bifunctional inhibitor of the SLC4 anion exchanger family containing two isothiocyano groups known to alkylate lysine residues of eAE1 [25,26] to those of Inh-172 (I-172), a CFTR blocker [10,13]. 

**Figure 1:**
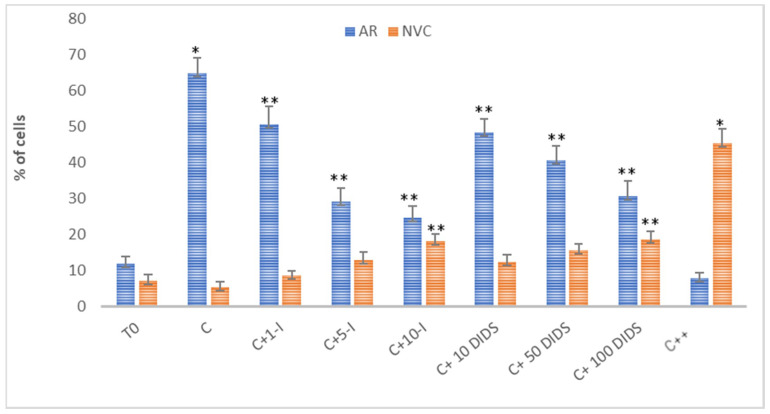
Effect of I-172 and DIDS on sperm capacitation and viability. Acrosome reaction induced by calcium ionophore A23187 and viability assay (tested with PI) at (T0) or incubated for 120 min in the absence (C) or presence of I-172 1, 5, 10 μM (C+1-I, C+5-I, C+10-I, respectively) or DIDS 10, 50, 100 μM (C+10 DIDS, C+50 DIDS, C+100 DIDS, respectively) or both (5 μM I-172 and 100 μM DIDS) (C++) were performed as described in Methods. Percentages of acrosome reacted cells (ARC%) and not viable cells (NVC%) were determined by immunofluorescence (see Methods). Comparison to both T0 and C was performed. Values represent the means ± S.D. of at least 8 experiments. For C compared to T0. *, *p* < 0.001; others compared to C, ** *p* < 0.05.

Notably, although all of the SLC4 family members AE1-3 are sensitive to DIDS, which is due to the sequence homology (53–56%) that these latter channels share [25], 70–100 μM DIDS is sufficient to block AE1 [26,27] as compared to the concentrations five [27,28] to 10 times higher [29] required to inhibit the other members of this channel family. Also, 5 μM I-172 has been described to completely inhibit CFTR and leave sperm viability unaffected, while reducing HCO_3_^−^ uptake and the PKA-related downstream pathways [10,13]. Taking all these considerations into account, sperm samples were incubated in the presence of increasing concentrations of HCO_3_^−^ blockers for 120 min, subsequently analyzing the response to the treatment as a percentage of acrosome reacted-cells (ARC%) and not viable cells (NVC%). As shown in Figure 1, both compounds induced a concentration-dependent decrease in the ARC%. 5 μM I-172 reduced sperm AR to 29 ± 3.8%, 10μM inducing an additional slight decrease (24.5 ± 3.3%), values dramatically lower than those measured with capacitated sperm (C 64.9 ± 4.3%, *p* < 0.05). However, I-172-dependent inhibition of the AR was not complete, ARC% remaining twice as high as T0 (11.8 ± 2.1, *p* < 0.001). On the other hand, even though not as efficiently, increasing concentrations of DIDS decreased ARC% significantly, with a trend similar to, albeit slightly higher than, that observed with I-172 (40.5 ± 4 and 30.5 ± 4.4, at 50 μM and 100 μM, respectively, compared to C, *p* < 0.001). When used in combination (5 μM I-127 and 100 μM DIDS), ARC% was reduced to 8%, a value even lower than that reached at T0 (*p* < 0.001, compared to C and T0).

Both inhibitors negatively affected cell viability, with NVC% reaching 18.1 ± 2 at 10 μM I-172 and 18.6 ± 2.2 at 100 μM DIDS (5.2 ± 1.5 upon C, and 7.1 ± 1.7 at T0, *p* < 0.05). Interestingly, a synergistic effect was observed when these molecules were used in combination, NVC% rising to 45.3 ± 4.1 compared to the controls (*p* < 0.001).

Consistent with previous studies [30], DIDS and I-172 also affected progressive and non-progressive motility, straight-line velocity (VSL), average path velocity (VAP) and amplitude of lateral head displacement (ALH) in a dose-dependent manner (Table S1). To avoid possible interference due to DIDS toxic effect on cell viability, we also performed experiments in parallel by incubating cells in a bicarbonate-free medium in the presence or absence of 100 μM DIDS over time (Supplementary Figure S1). As expected, no increase in ARC% was observed, this medium proving unable to induce capacitation in the absence of bicarbonate. In addition, the increase in NVC% in a time-dependent manner was observed independently of the presence of DIDS, confirming that it was the absence of bicarbonate to affect cell viability.

#### 2.1.2. Effect of I-172 and DIDS on Tyr-P Level and Location as well as Membrane Rearrangement

**Figure 2:**
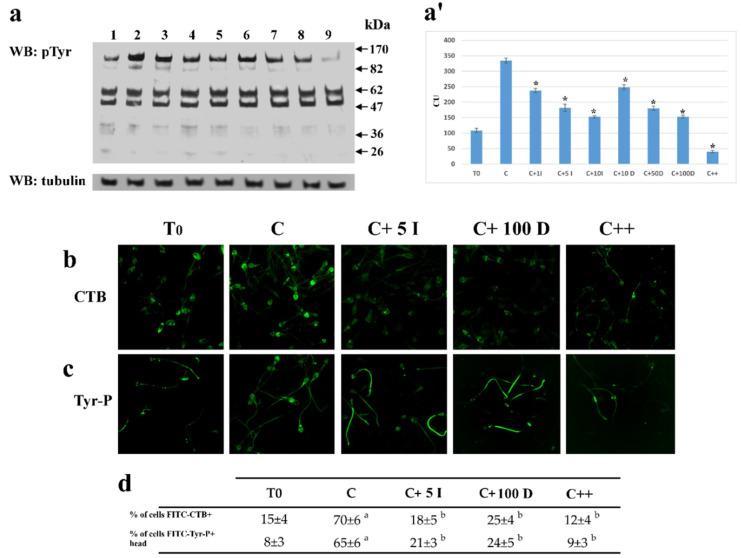
Effect of I-172 and DIDS on sperm Tyr-P level and CTB displacement. (**a**): Sperm samples (1 × 10^6^ cells) before, T0 (lane 1), and after 120 min of incubation in the absence (lane 2) or presence of 1 (line 3), 5 (lane 4) 10 (lane 5) μM I-172, or 10 (lane 6), 50 (line 7) or 100 (lane 8) μM DIDS, or both (lane 9) were analyzed by Western blotting with anti-P-Tyr antibodies, the response to which was normalized against tubulin expression; all as described in Methods); (**a’**) bands corresponding to the phosphorylated proteins were densitometrically estimated, normalized to tubulin, and statistically analyzed. The figure is representative of 6 separate experiments. Data show the means ± SD of relative units (RU). Comparison to C values: * *p* < 0.001, (**b**). FITC-labeled CTB staining (panel **b**), highlighting the shifting of ganglioside GM1 embedded in membrane microdomains, and FITC-labeled P-Tyr staining (panel **c)** on sperm cells were also analyzed by immunofluorescence in T0, C, C+5μM I-172 (C + 5I), C+100μM DIDS (C+ 100 D), or both (C++), as described in Methods. The figure is representative of 3 separate experiments. (**d**) Number of cells FITC-CTB + and number of cells Tyr-P + in the head are expressed as means ± SD% of the total number of cells analyzed. (a *p* < 0.0001, b: *p* < 0.001, comparing each sample against C values).

As the progression of capacitation is connected with to the Tyr-P level [19,20,29,31], samples incubated as above were analyzed for Tyr-P levels in total cell lysates (Figure 2).

Both I-172 (5 μM) and DIDS (50 μM) reduced Tyr-P in the total cell lysate by 50% as compared to C (181 ± 12 and 180 ± 6.6, compared to 334.5 ± 7.6 RU, respectively, *p* < 0.001) with a slight further decrease at higher concentrations, but never dropping to the T_0_ levels (108.4 ± 6.9). When used in combination, the compounds almost completely abrogated Tyr-P, which was less than half compared to T0 (40.2 ± 3.9, *p* < 0.001) (Figure 2, panel a, a’).

In order to evaluate the effect of inhibitors on the capacitation-related membrane rearrangement and Tyr-P-level, sperm cells, capacitated in the absence (C) or presence of either inhibitor or both, were analyzed by FITC-labeled Cholera Toxin B (CTB) subunit (panel b) and FITC-Tyr-P labeled cells (panel c), respectively. Either inhibitor reduced membrane rearrangement and the Tyr-P levels of the apical part of the sperm.

### 2.2. Identification and Localization of Sperm SLC4A1 (spAE1) and Protein Kinase Syk

**Figure 3:**
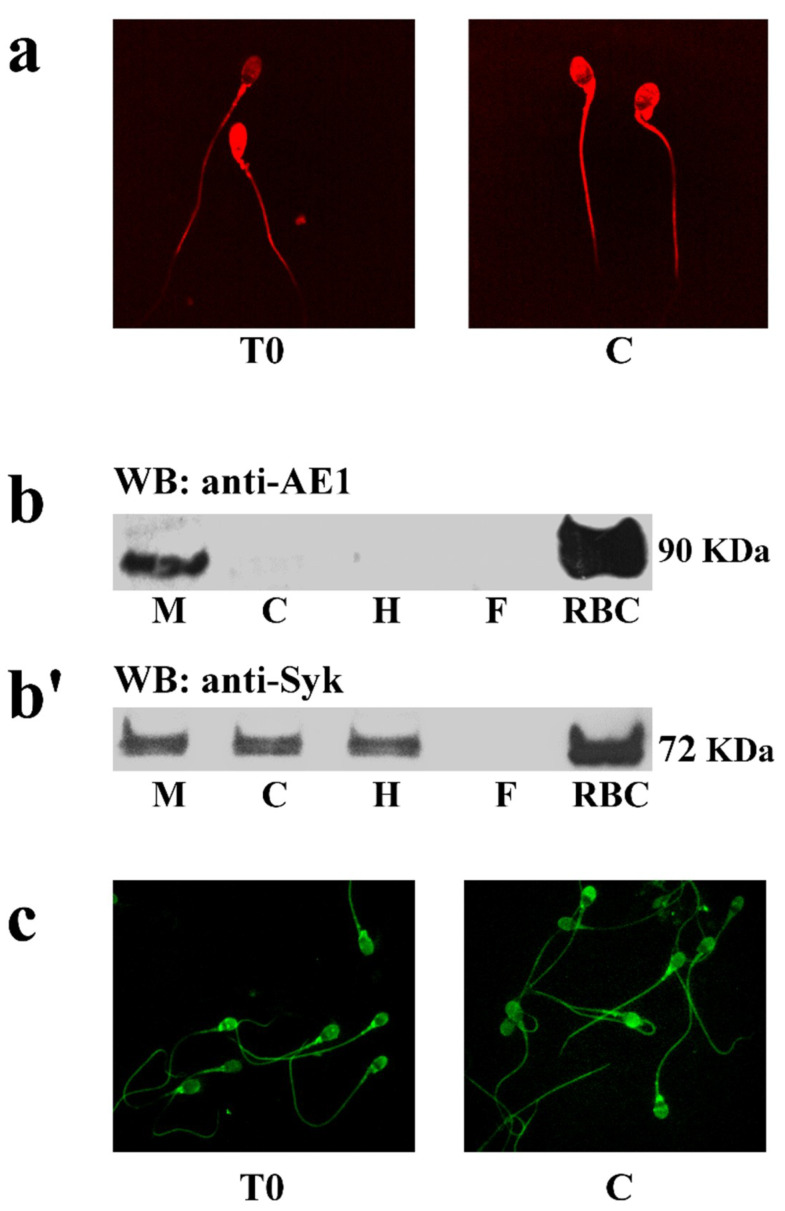
Expression and location of spAE1 and protein kinase Syk. Panel (**a**) Immunofluorescence of spAE1 in sperm incubated under non-capacitating (T0) or capacitating (C) conditions. (**b**) Western blot analysis of the subcellular sperm fractions obtained as described in Methods. Non-capacitated sperm plasma membrane (M), cytosol (C), flagellum (F) or head (H) were subjected to Western blot analysis with antibodies against spAE1 (panel (**b**)) or Syk (panel (**b’**)). Panel (**c**) Immunofluorescence detection of protein kinase Syk in sperm incubated under non-capacitating (T0) or capacitating (C) conditions. The figure is representative of 6 separate experiment.

To assess the expression and location of spAE1, sperm cells were analyzed by immunofluorescence (Figure 3), with an antibody directed against a 20 kDa epitope of the N-terminus of human AE1 (also known as band 3).

Figure 3a shows that AE1 is expressed throughout the sperm membrane with no detectable changes between T0 and C. Unexpectedly, Western blot analysis of total cell lysates did not reveal the presence of AE1, likely because of the elevated content of the fibrous sheath of flagella and DNA strands, which prevented the correct detection (data not shown). Following subcellular fractionation, spAE1 was identified in the membrane compartment, with a net band with an apparent molecular mass of 90kD, corresponding to the low border of the diffuse band of the eAE1 used as positive control (Figure 3b).

In human RBCs, the regulation of eAE1 function involves Tyr-P, which relies on the activity of two different tyrosine kinases, Syk and Lyn. Since Lyn has already been identified and characterized in sperm cells [23], blots were only probed with anti-Syk antibodies (panel b’), and the tyrosine kinase Syk was detected in the membrane (M), cytosol (C), and flagella, but not in the head (H). In addition, Syk was detected all over the cell by immunofluorescence, with no significant changes between T0 and C (panel c).

### 2.3. Relationship between spAE1 Tyr-P and Capacitation

#### 2.3.1. Effect of Capacitation and I-172 or DIDS on the Tyr-P Level of spAE1 and spAE1’s Interaction with Syk

**Figure 4:**
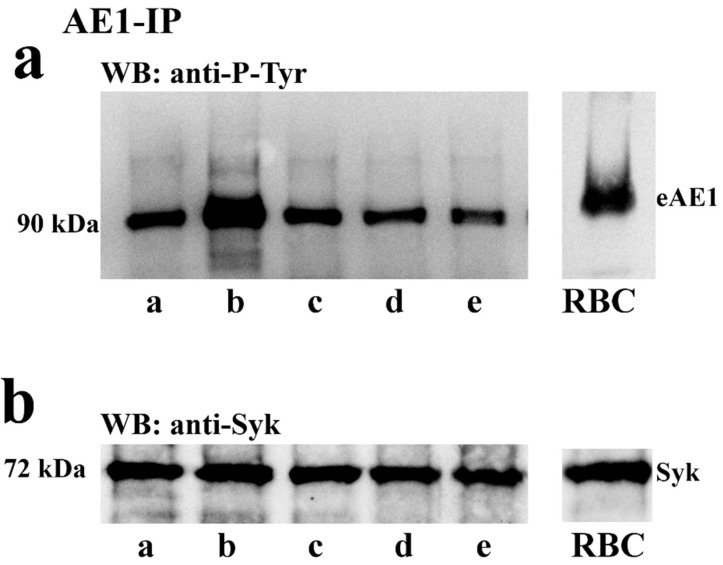
Sperm cells were either immediately processed (T0, lane a) or incubated for 120 min in the absence (C, lane b) or presence of +5 μM I-172 (C+5I, lane c), 100 μM DIDS C+DIDS, (lane d) or both (C++, lane e). Samples were extracted and immunoprecipitated with anti AE1 antibody and immunoblotted with anti-P-Tyr (panel (**a**)) or anti-Syk (panel (**b**)) antibodies. The RBC lane shows the anti-P-Tyr staining of erythrocyte membrane. The figure is representative of 6 separate experiments.

To assess whether the phosphorylation of tyrosine residues of spAE1 was regulated by the capacitation process, spAE1 was immunoprecipitated from cell lysates of sperm samples treated with I-172 or DIDS, the CFTR and AE1 blockers described above, respectively. When spAE1-IP were analyzed by immunoblotting (Figure 4a) a 90 kDa band was detected by anti-P-Tyr antibodies, which corresponded to spAE1 when re-probed by anti-AE1 antibodies (data not shown). This P-spAE1 band was markedly increased under capacitating conditions (lane b) (754 ± 45 compared to 253 ± 8.5 in T0, *p* < 0.05, Supplementary Table S2), but dropped to levels similar to those observed at T0 in sperm cells previously treated with I-172 (225 ± 12) and even lower with DIDS (204 ± 11), or both (115 ± 34).

Figure 4b shows that Syk co-immunoprecipitated with spAE1 in the spAE1-IP, which was further confirmed by co-immunoprecipitation of spAE1 in the Syk-IP assay (Supplementary Figure S3). On the other hand, treatment with I-172 and DIDS did not alter the interaction between the two proteins, the amount of both remaining stable in all the immunoprecipitated samples, even if the spAE1-IP was Tyr-phosphorylated to different extents. Moreover, when Syk-IPs were probed with anti-P-Tyr antibodies, a 90 kDa band was revealed which increased in intensity in sample C, however declining to the T0 levels when sperm samples had been treated with I-172 or DIDS, or both (Supplementary Figure S2 and Table S2).

Similar experiments were also carried out to assess whether Lyn co-immunoprecipitated with spAE1, which failed to reveal an association between the two proteins (data not shown).

#### 2.3.2. Effect of SykI and LynI on Capacitation (Tyr-P, CTB, P-AE1) and AR

To analyze the regulation of spAE1 Tyr-P process, aliquots of sperm samples incubated in the presence of either Syk or Lyn inhibitors were analyzed by immunofluorescence with an antibody directed against the phosphorylated form of Tyr-359 (pTyr359 AE1, or P-AE1) of eAE1, which is targeted by Lyn, and can be used to discriminate between the activities of Syk and Lyn and their involvement in the capacitation process (Figure 4, panel c).

Interestingly, spAE1 P-Tyr359 was weakly diffuse throughout the sperm (T0), with a net increase of fluorescence in C samples, mainly in the head region (C) (Table 1). Surprisingly, the Syk inhibitor (SykI, 1 μM) induced a net increase in fluorescence, especially in the sperm head, which was totally abrogated after incubation with the Lyn inhibitor (LynI, 1 μM). Similarly, spAE1 P-Tyr359 was also suppressed by incubating sperm with both inhibitors (+SykI +LynI). 

**Figure 5:**
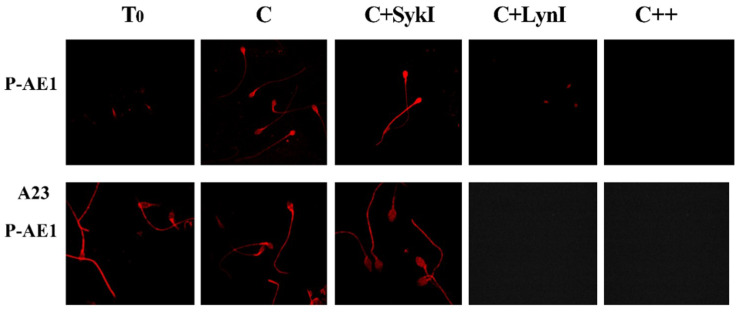
Selective phosphorylation of spAE1 on Tyr359 residue. Sperm cells, isolated and treated for 120 min under capacitating conditions in the absence (C) or presence of protein kinase Syk (SykI) or Lyn (LynI) inhibitors, were analyzed at the end of incubation (P-AE1) or after further incubation for 30 min with A23187 (A23 P-AE1) for the AR. Immunofluorescence with anti-P-AE1 antibody was performed as described in Methods. Panels are representative of 6 separate experiments.

Aliquots of sperm, incubated as described above, were also treated with A23187 (calcium ionophore) for 30‘, in order to induce AR in properly capacitated sperm (Figure 5, panel A23). Interestingly, the anti-AE1 P-359 labelling was evident in the sperm head in T0, C, and SykI samples more markedly than the corresponding samples before A23 treatment (Figure 5, panel AE1), but not in samples incubated with LynI (panel A23, P-AE1).

When the effect of SykI and LynI was tested on sperm function, aliquots of sperm treated as described above were analyzed for sperm membrane rearrangement, Tyr-P, the ability to undergo AR and viability. Furthermore, the percentage of not-viable cells (NVC%) present in the samples was also measured after each treatment.

Capacitation involves membrane remodeling, monitored by CTB-labeled membrane microdomains accumulating to the sperm head [22,32]. Also, the tyrosine kinase Lyn, embedded in these membrane microdomains, is dragged to the head region where it induces the increase of Tyr-P level in the sperm head [23]. The results reported in Table 1 show that the CTB labelling was partially inhibited by SykI (55.5 ± 3.5 compared to C 67 ± 3.6, *p* < 0.05), which was also the case of the percentage of cells undergoing Tyr-P in the sperm head (58.7 ± 2.3 compared to C 66.3 ± 2.9, *p* < 0.05), We also observed a net increase of the percentage of cells stained with anti p-Tyr359 antibody in the head, which was even higher than in C (63.2 ± 2.5, *p* < 0.05) but with ARC% showing a slight decrease as compared to C (52.9 ± 3.2 vs. 69.7 ± 5.2, *p* < 0.001). LynI, in turn, caused Tyr-P in the sperm head, CTB and P-Tyr359 to drastically drop to 21.3 ± 1.7, 23.3 ± 4.2, and 16.3 ± 3.5, respectively (*p* < 0.001) along with a net impairment of the AR (ARC 20.4 ± 4.1, *p* < 0.001 compared to C), with a net increase of NVC% (60.6 ± 1.3 vs. C 9.1 ± 1.2, *p* < 0.05)

When used in combination, all these parameters further dropped, with a net increase of NVC% (65.4 ± 3.2% compared to C, 9.1 ± 1.2%, *p* < 0.05).

These data confirm the role of spAE1 Tyr-P in the progression of capacitation and in cell survival.

It can be hypothesized that Syk recruitment to spAE1 is regulated either by the presence of a protein/phospholipid mediating the interaction between the two proteins, or by constitutive Tyr-P already present in sperm at T0, as a sufficient pre-requisite to a direct binding between the Syk SH2 domains and one of the spAE1 phosphorylated Tyr-residues. This latter would be the most probable hypothesis because spAE1 never undergoes total dephosphorylation, even when samples were treated with SykI and LynI (Figure 2, panels a and c).

#### 2.3.3. Evaluation of Sperm Motility

Capacitation–related motility parameters, such as increase in straight-line velocity (VSL), average path velocity (VAP) and amplitude of lateral head displacement (ALH) were evaluated under different conditions and after treatment with SykI or LynI (Table 2). All such parameters remained quite similar to C after all the treatments, which ruled out a direct involvement of Syk or Lyn in the regulation of sperm motility.

## 3. Discussion

In the present study, we demonstrated that spAE1 is expressed and distributed in the membrane of human sperm cells, actively contributing to the viability and capacitation, as demonstrated by the increased NVC% and decreased ARC% when sperm cells were treated with DIDS, an inhibitor of AE1-mediated anion exchange (Figure 1). 

Previous studies showed that specific inhibitors of CFTR and the Na/HCO_3_^−^ cotransporter (NBC), or the epithelial Na-channels (ENaCs) blockers [9,24] inhibited flagellar movements, capacitation and AR, but also reduced the extent of Tyr-P, implying a role of bicarbonate in sperm Tyr-P.

Our results not only confirm the bicarbonate-related modulation of the Tyr-P process, but also corroborate the notion that, rather than the total amount of proteins, the location of proteins undergoing Tyr-P in the apical part of the cells is essential for the subsequent AR [18].

An interesting study on ionic currents monitored in the whole cell of mature human spermatozoa showed a net inhibition of the AR induced by DIDS, occurring almost completely at a concentration as low as 50 μM [25]. In our experiments, even at 100 μM, DIDS never abolished AR (Figure 1), which instead was observed when added also in the presence of I-172, a CFTR inhibitor. This discrepancy may be due to the sperm capacitating conditions of our experiments, leading to large number of cells reaching the AR (more than 60%), compared to almost half in the previous study. So far, we can assume that CFTR and spAE1 cooperate in maintaining the uptake of bicarbonate. Inhibition of either of them results in a partial impairment of the capacitation process by hindering membrane rearrangement and reducing the level of Tyr-P in the head of the cells, consequently preventing sperm cells from undergoing AR.

The direct involvement of spAE1 Tyr-P in the various sperm processes is further demonstrated by its inhibition, which reduces both viability and capacitation. As the AE1 isoform of the SLC 4 family has been identified and characterized mainly in the red cell membranes, including the role of Tyr-P in its structural and functional behaviour, we have assumed that Tyr-P affects spAE1 in a similar manner. First, we highlighted that Syk (spleen tyrosine kinase), the prototypical member of the homonymous protein kinase family, is present in membranes, but also in the cytosol and head of sperm cells but not in flagella, as in erythrocytes where Syk is found as both a soluble and membrane-bound form. Second, as evidenced in erythrocytes, Syk strongly interacts with spAE1 (as indicated by reciprocal co-immunoprecipitation) and catalyzes the Tyr-P of specific tyrosine residues. Third, unlike the erythrocyte model, Syk-mediated phosphorylation of spAE1 is not essential for Lyn recruitment and subsequent phosphorylation of Tyr359 [17]. In fact, Syk inhibition increases the phosphorylation of Tyr359 in human sperm, supporting the hypothesis that the active form of Syk could hamper Lyn activation rather than sequentially induce it. The different behavior of Lyn in sperm cells and RBCs relies on the different membrane structures. Indeed, in human erythrocytes Lyn is stably embedded in the membranes and can relocate only through the Lyn-SH2 recruitment to eAE1-P-Tyr. Conversely, in human sperm, Lyn, embedded in membrane microdomains, reaches its substrate more easily, being relocated by raft shifting during capacitation-related membrane reorganization [23]. This shifting allows Lyn to target spAE1 directly, without being recruited through P-Tyr-SH2 interaction. It can also be hypothesized that, opposite to eAE1, the other Tyr residues, when phosphorylated by Syk, may sterically prevent Try359 phosphorylation, at least partially, as can be inferred by the higher level of phosphorylation of Tyr359 resulting from Syk inhibition, especially in the head, compared to C. This increase of P-Tyr359 is related to an increase of ARC and suggests a direct role of this tyrosine residue in preparing the cell for, and an indirect role of Syk in part preventing, the AR. Only a slight impairment of both AR and viability is caused by Syk inhibition, whereas Lyn inhibition brings about a high increase in NVC% and decrease in ARC%. The relevance of the Lyn-catalyzed Tyr-P of the Tyr359 of spAE1 (Figure 5) is underscored by the effects of its inhibition which prevents both capacitation and viability (Table 1). Taking together, we can assume that spAE1 Tyr-P is required to support both the capacitation-related membrane rearrangement leading to AR (as evidenced by the CTB and ARC percentages) and viability (NVC%) (Table 1). In addition, the phosphorylation of Tyr359 is involved in the AR, as indicated by the close relationship between the percentages of cells presenting P-Tyr359 and AR, both drastically declining in the presence of LynI, but not of SykI, this latter blocking neither capacitation nor AR. The increased level of P-Tyr359 fluorescence during AR (Figure 5, panel A23) further confirmed the involvement of P359-AE1 also in this final step.

Even though the combination of SykI and LynI markedly reduced viability (increased NVC%), motility was only slightly, if at all, affected (Table 2), once more confirming that motility and viability, conceived as nuclear integrity, are not strictly related [29,31,33], and the evaluation of the simple motility parameter may be misleading in the determination of the sperm/semen quality. 

The need for bicarbonate for sperm cell function has been widely demonstrated, CFTR proving to be the essential factor responsible for its uptake, whereas some doubt exists as to the occurrence of an additional channel that causes bicarbonate to enter sperm cell initially [10,13]. Based upon results reported here, it can be inferred that: (i) CFTR alone, as well as the spAE1 alone, proved to be only in part responsible for the bicarbonate-dependent mechanism leading to the capacitation, thus supporting a previous hypothesis of one or multiple channels cooperating for the entry of HCO_3_^−^, and (ii) bicarbonate concentration is not only important for the progression of sperm, but also for cell survival, as shown by the elevated NVC% when both channels are blocked.

CFTR function depends on a conformational change, driven by ATP binding and hydrolysis, shifting the molecule to the open state. This change, triggered by PKA-dependent phosphorylation of the CFTR regulatory domain, is stimulated by a rise of cAMP, the PKA activator, which depends on the HCO_3_^−^ increase [13]. The occurrence of an additional channel, not related to CFTR, was postulated to explain this initial HCO_3_^−^ uptake which would initiate all the downstream events [13,34]. Previous studies also showed that Tyr-P decreased in the presence of either specific inhibitors of CFTR, or blockers of the Na/HCO_3_ cotransporter (NBC) and Na-channels (ENaCs) [9,24], corroborating the notion of a strong relationship between bicarbonate entry and phosphorylation in capacitation. 

The eAE1 exchanger activity is up-regulated by casein kinase 2-dependent phosphorylation of the serine residues in the cytoplasmic domain of the protein [35,36,37], whereas the phosphorylation of its tyrosine residues affects the interactions with other proteins and enzymes [15,16]. In particular, Tyr-P triggers eAE1′s conformational changes in the N-cytoplasmic terminus, in turn promoting the dissociation of band 3 from the cytoskeleton, especially the spectrin–actin network due to a decrease in affinity to ankyrin [38] by triggering eAE1′s conformational changes in the N-cytoplasmic terminus, in turn leading to the dissociation of eAE1 itself from the spectrin–actin network due to a decrease in affinity to ankyrin [38]. In human sperm, actin has been found to act as a regulatory protein in the complex cytoskeletal rearrangement leading to the opening of the acrosomal region. An increase in F-actin (actin polymerization) during capacitation is necessary to sustain the fibrous sheath for the hyperactivated motility and to create a network in the sperm head between the plasma membrane and the outer acrosomal membrane. Prior to AR, the dispersion of this F-actin (actin depolymerization) through a fast F-actin breakdown must occur to elicit the acrosomal opening and exocytosis [39]. The increase in the spAE1 Tyr-P level in the apical part of the cell (head/acrosome) may be explained in light of the role of Tyr-P in the protein/protein and lipid/protein interactions occurring between the plasma membrane and the cytoskeleton. 

Further studies are certainly warranted to better understand the impact of the aberrancies of the spAE1 Tyr-P level on the regulation of male fertility. Whereas the role of bicarbonate in male infertility has been studied for many years [40], the data remain inconclusive and controversial as to the role of Tyr-P level. We have previously shown that the activation of Lyn in a group of patients with idiopathic infertility was lower when compared to controls [23], thus further confirming the key role played by Lyn-mediated Tyr-P in the capacitation progress and fertilization. Since Lyn is the enzyme that catalyzes the phosphorylation of spAE1 Tyr359-P, which is strictly related to both capacitation and AR, investigations focused on the Tyr-P level of this protein would be helpful to identify new landscapes for the treatment of infertility.

Whatever the role of the Syk-mediated Tyr-P in the regulation of the capacitation process, the level of Tyr-P of the sperm head and of spAE1, at Tyr359 in particular, are strongly related to sperm AR, indicating its physiological role in fertilization.

## 4. Materials and Methods

### 4.1. Semen Collection and Analysis

Twenty-four healthy male donors of proven fertility (with 1–3 children) (age range: 24–37 years, average age: 31.4 years) were enrolled at the Department of Medicine-Endocrinology, University of Padova, Italy. After 3 days of abstinence, semen samples were collected by masturbation in a sterile container and then assessed for sperm parameters. All sperm samples used in this study were normal in terms of sperm count, motility, morphology, volume, fructose level, and pH, according to the World Health Organization criteria [41]. All samples presenting any kind of contamination were discarded. This study was approved by the Ethics Committee for Research and Clinical Trials of our University (02-13-2012), and all recruited donors gave their informed written consent and provided detailed lifestyle histories.

### 4.2. Chemicals

Mouse monoclonal anti-P-Tyr (Clone Py20), goat anti-Mouse IgG-fluorochrome fluorescein isothiocyanate (FITC) conjugate and anti-Rabbit Alexa fluor 594 antibodies were purchased from Upstate (Becton Dickinson Italia SpA, Milan, Italy), Santa Cruz Biotechnology (Heidelberg, Germany), and Thermo Fisher Scientific (MI, Italy), respectively. Rabbit monoclonal anti-Anti-Band 3/AE 1 (phospho Y359) antibody was obtained from Abcam (Cambridge, UK) and other secondary antibodies from Santa Cruz Biotechnology (Heidelberg, Germany). Density gradient (Pure Sperm 40/80) and capacitating pure sperm wash (PSW) buffer were obtained from Nidacon International AB (Göteborg, Sweden). 4,4′-Diisothiocyanostilbene-2,2′-disulfonic acid (DIDS) and CFTR_inh_-172 were from Calbiochem (La Jolla, CA, USA), 2,3-dihydro-3-[(1-methyl-1H-indol-3-yl)methylene]-2-oxo-1H-indole-5-sulfonamide (Syk Inhibitor) from Cayman Chemical (Ann Arbor, MI, USA) and Saracatinib (Lyn Inhibitor) from Sellekchem (Houston, TX, USA). Mouse monoclonal anti-Band 3 (AE1) clone BIII-136 ascites fluid, anti-tubulin, and all other reagents were from Sigma-Aldrich (Milan, Italy).

### 4.3. Sample Preparation

After semen analysis, samples were laid on a discontinuous gradient (Pure Sperm 40/80%) and centrifuged at 500× *g* for 30 min at room temperature. The seminal plasma and sperm from the 40% gradient interface were discarded, and the sperm cells from the bottom pellet (80% gradient) were gathered. After gradient separation, sperm samples were collected three by three in a single pool to obtain enough cells to perform all tests (AR status and viability, Tyr-P) for each experiment. Cells were washed in capacitating medium (PSW), re-analysed for concentration, motility, viability, and morphology, and collected in a single vial (stock sample) at a concentration adjusted to 80 × 10^6^ sperm cells ⁄mL in PSW. The chloride channel blockers, I-172 and DIDS were both prepared as stock solutions of 100 mM in dimethylsulfoxide (DMSO) and diluted to final concentrations in PSW sperm suspension. DMSO controls were included in the experiments. The concentrations of I-172 and DIDS were chosen based on previous studies [13,30] Also, kinase inhibitors, SykInhibitor (SykI) or Saracatinib (LynI), dissolved in 100 mM in DMSO, were added to PSW sperm suspension to 1 μM final concentration [42] following manufacturer’s indications.

Stock sperm samples from different treatments were prepared and aliquots analyzed immediately (T_0_) or incubated for up to 120 min in capacitating conditions (PSW), in the absence (C) or presence of increasing concentrations of I-172 (1, 5 or 10 μM) or DIDS (10, 50, or 100 μM), or both (5 μM I-172 plus 100 μM DIDS). Similar experiments were also carried out in non-capacitating medium containing NaCl 110 mM, KCl 4.80 mM, CaCl_2_ 1.68 mM, MgSO_4_ 1.19 mM, KH_2_PO_4_ 1.17 mM, sodium pyruvate 0.25 mM, glucose 5.48 mM, sodium lactate 25 mM, Hepes 10 mM, and 4 mg/mL bovine serum albumin (BSA).

### 4.4. Computer Assisted Sperm Analysis (CASA)

Sperm motility and hyperactivation were analyzed using an IVOS II Clinical computer-assisted sperm analyzer (CASA) with Human Motility software.

For each sample, the following parameters were evaluated: the percentage of motile spermatozoa and VCL (curvilinear velocity), VAP (average path velocity), VSL (straight-line velocity), and ALH (amplitude of lateral head displacement) to determine the percentage of motile cells. Only cells with VCL ≥150 µm/s, LIN (VSL/VCL) ≤50%, and ALH ≥7 µm [36] were considered. All measurements were performed at 37 °C. A minimum of 100 cells and 5 fields were analysed for each aliquot [38].

### 4.5. Anti-Tyr-P and Anti-P-AE1 and Anti-Syk Immunofluorescence Evaluations 

Aliquots of sperm (15 × 10^6^ cells) from each sample were washed with phosphate buffer saline (PBS) containing vanadate 1 mM and protease inhibitor cocktail, fixed with 2% (*w*/*v*) paraformaldehyde and incubated overnight at 4 °C on slides pre-coated with poly-L-lysine [19]. Slides were rinsed twice with PBS and sperm cells were permeabilized with 0.2% (*v*/*v*) Triton X-100 for 15 min at 4 °C, blocked with 1% BSA for 30′ at RT and incubated with specific antibodies according to the manufactory’s instructions (anti-P-Tyr diluted 1:20 in 1% bovine serum albumin (BSA) in PBS, anti-P-AE1 diluted for 1 h at 37 °C in a wet chamber). Slides were washed three times with PBS and then stained with anti-Mouse IgG-FITC conjugated or Rabbit Alexa-fluor 594 (both diluted 1:100 in 1% BSA in PBS) for 1 h at 37 °C in a wet chamber, rinsed with PBS and mounted. Staining without primary antibody was used as negative control. Fluorescence was detected with the UltraView LCI confocal system (Perkin Elmer, Waltham, MA, USA) equipped with a fluorescence filter set for excitation at 488 or 594 nm.

At least 200 cells were evaluated for each sample, and fluorescence detected on the head of cells (Tye-P and P-AE1) or spread (Syk) was recorded.

### 4.6. Evaluation of A23187-Induced Acrosome Reaction and Sperm Viability

Acrosome status was monitored with acrosome-specific FITC-labelled peanut (*Arachis hypogaea*) agglutinin (FITC-PNA) in conjunction with DNA-specific fluorochrome intercalant propidium iodide (PI), which is commonly used to detect dead cells in a population. In fact, acrosome reaction implies the breakdown of the external acrosomal membrane and the fusion of the internal acrosome membrane with the underlying plasma membrane in the absence of membrane damage, thus preventing PI, being not permeant to intact membranes, from binding to DNA [23,43,44].

For this reason, propidium iodide cannot reach and bind to nuclear DNA and can be commonly used to discriminate viable from not viable cells in tests dosing the acrosome reacted cells.

Briefly, in order to induce AR, aliquots (15 × 10^6^ cells) of each sample were incubated for 30 min at 37 °C, in the presence of 10 µM Ca^2+^ ionophore A23187 [19]. Samples containing DMSO, but not ionophore, were used as control. After incubation cells were centrifuged, resuspended in PBS and treated for 10 min at room temperature with 12 μM PI. Sperm was washed with PBS, fixed with 2% (*w*/*v*) paraformaldehyde and incubated overnight at 4 °C on poly-l-lysine-treated slides. After permeabilization in 0.2% (*v*/*v*) Triton X-100, sperm cells were stained with 1 mg FITC-PNA ⁄mL for 15 min at 37 °C in the dark, washed and mounted. At least 200 cells were evaluated for each sample, and fluorescence was detected as described above. Only sperm cells with no or very low red fluorescence were considered as viable. Cells showing evenly distributed fluorescence over the acrosomal region were considered acrosome-intact or as acrosome reacted when either fluorescent staining was restricted to the equatorial segment or no labeling was observed.

### 4.7. Evaluation of Membrane Rearrangement

The ganglioside GM1, which is a marker of membrane microdomains [45], was visualized in live human spermatozoa by staining with the cholera toxin subunit B (CTB)-FITC [13,14]. For this purpose, suspensions of cells (15 × 10^6^ cells) from each sample were mixed with an equal volume of CTB (50 µg/mL) and incubated for 15 min at 37 °C. The sperm cells were then washed twice in PBS before being fixed in 2% paraformaldehyde for 30 min, mounted on poly-L-lysine coated glass microscope slides, and viewed using the confocal microscope as described above. For each treatment, at least 200 cells were counted and categorized into four different fluorescent patterns (acrosome, sub-acrosome region, neck and tail). 

### 4.8. sp AE1 and Syk Localization and Interaction

#### 4.8.1. Anti-AE1 (sp/eAE1-IP) and Anti-Syk Immunoprecipitations

Sperm cells (20 × 10^6^) from each sample were resuspended in 300 µL of PBS (containing 1 mM sodium orthovanadate and protease inhibitor cocktail). Thirty µL (corresponding to about 2 × 10^6^ cells) were used for Western blotting, and treated with 2 mM (final concentration) of 3-[(3-cholamidopropyl) dimethylammonio]-1-propanesulphonate hydrate (CHAPS) [19] at 0 °C for 10 min.

The remaining volume of cells resuspended in PBS (containing 1 mM sodium orthovanadate and protease inhibitor cocktail) (see above), was extracted in Igepal buffer (Igepal 1% final concentration, Tris-HCl 20 mM, EDTA 2 mM, NaCl 300 mM EGTA 10 mM, 1 mM sodium orthovanadate and protease inhibitor cocktail) at 4 °C for 2h in agitation. After centrifugation, supernatants were pre-cleared with protein A-Sepharose, and then incubated overnight at 4 °C with anti-AE1 (3–5 μL) or anti Syk (3–5 μL) antibody in agitation and recovered by protein A Sepharose addition. Immune complexes were washed three times in 50 mM Tris–HCl pH 7.5, 1 mM vanadate and protease inhibitor cocktail, and subjected to Western blotting. 

To obtain eAE1-IPs as a comparison, packed erythrocytes (1 mL) from healthy volunteers were hemolyzed in 10 volumes of hypotonic phosphate buffer 10 mM pH 8 and pelleted membranes were extracted, immunoprecipitated and analyzed as described above.

#### 4.8.2. spAE1 Localization in Sub-Cellular Fractions

To determine whether spAE1 was present in the plasma membrane, head or flagellum, intact spermatozoa (3 × 10^7^ cells) were sonicated 3 times (30 s followed by a 30-s rest period each) on ice, and heads and flagellar fragments were then separated by a 15-min centrifugation (700× *g*) at 4 °C through a 75% Percoll layer in PSW. The use of sonication induces the breaking of plasma membranes which, during centrifugation, remained in the supernatant with the cytosol, whereas tail fragments were recovered at the surface of the Percoll layer and the heads were found in the pellet [46].

The purity of each fraction was assessed by microscopy prior to proceeding to analysis. The supernatant was centrifuged for 10 min (10,000× *g*, 4 °C) and the resulting supernatant was further centrifuged (100,000× *g*) to separate the membrane from the cytosol [22]. Each resulting fraction (membranes M, cytosol C, head H and flagella F) was diluted with PBS to the initial volume except for membranes which were concentrated three times, and further processed as described above for total cell lysate. The presence of AE1 and Syk was investigated by Western blotting and immuno-revealed with anti-AE1 antibody.

The nitrocellulose blots (Millipore, Bedford, MA, USA) were blocked for 1 h at RT with 3% bovine serum albumin (BSA) in tris-buffered saline (TBS-T buffer) containing 100 mM Tris-HCl, pH 7.5, 150 mM NaCl, 0.2% Tween 20), probed with specific antibody (following manufacturer’s recommended dilutions) for 1 h, rinsed 3 times in TBS, exposed to the HRP-conjugated mouse or rabbit secondary antibody for 30 min, rinsed 3 times in TBS, and detected by enhanced chemiluminescence (ECL by GE Healthcare UK Limited. Buckinghamshire, UK) with a Signal Kodak Image Station 4000 mm Pro Digital System (Eastman Kodak, Rochester, NY, USA). Successively, bands were counted by ImageJ free software and normalized against the housekeeping protein tubulin band.

### 4.9. Statistical Analysis

Results are expressed as means ± SD. Comparisons were obtained with Student’s *t*-test for paired or unpaired data and statistical significance was set at *p* < 0.05 (two-tailed). All statistical analyses were performed with JMP^®^ 10 software (SAS Institute, Cary, NC, USA).

## Figures and Tables

**Table 1:** Sperm biochemical parameters observed in different samples. Parameters were evaluated with fluorescence microscopy at T_0_ (before starting incubation) and after 120 min of incubation in capacitating conditions in absence (C) or presence of SykI (1µM), LynI (1μM) or both (C++) as described in Methods. Number of cells presenting FITC-CTB + or, Tyr-P + or P-AE1 in the head are expressed as means ± SD% of the total number of cells analyzed. P-AE1 was tested also at the end of further incubation with calcium ionophore A23187 (A23-P-AE1).

	Tyr-P (%)	CTB (%)	P-AE1 (%)	A23-P-AE1 (%)	ARC (%)	NVC (%)
	Head	Head	Head	Head		
T_0_	18.2 ± 2.1	15.4 ± 1.7	9.2 ± 1.2	10.8 ± 1.2	11.3 ± 2.3	7.1 ± 2.3
C	66.3 ± 2.9 *	67.0 ± 3.6 *	63.1 ± 2.5 *	68.9 ± 7.5 *	69.7 ± 5.2 *	9.1 ± 1.2 *
C+SykI	58.7 ± 2.3 ^b^	55.5 ± 3.5 ^b^	85.5 ± 12.7 ^b^	89.7 ± 7.7 ^b^	52.9 ± 3.2 ^a^	12.3 ± 1.1 ^b^
C+LynI	21.3 ± 1.7 ^a^	23.3 ± 4.2 ^a^	16.3 ± 3.5 ^a^	15.3 ± 4.6 ^a^	20.4 ± 4.1 ^a^	60.6 ± 1.3 ^b^
C++	10.2 ± 1.3 ^a^	12.8 ± 3.5 ^a^	9.9 ± 2.2 ^a^	8.5 ± 4.2 ^a^	14.6 ± 3.3 ^a^	65.4 ± 3.2 ^b^

^a^*p* < 0.001, ^b^ *p* < 0.05 comparison between various samples vs. C as reference; Student’s *t*-test for paired data. * *p* < 0.001; comparison C vs. T_0_; Student’s *t*-test for paired data.

**Table 2:** Sperm motility and kinematic parameters observed in different samples, evaluated with computer-assisted sperm analysis (CASA) at T_0_ (before starting incubation) and after 120 min of incubation in capacitating conditions in absence (C) or presence of SykI, LynI, or both. Motility = progressive and non-progressive motility (%); VSL = straight-line velocity (µm/s); VAP = average path velocity (µm/s); ALH = amplitude of lateral head displacement (µm).

	Motility (%)	VSL (µm/s)	VAP (µm/s)	ALH (µm)
T_0_	69 ± 3	58.4 ± 8.9	54.0 ± 6.7	3.1 ± 0.5
C	76 ± 7	77.8 ± 13.9 ^a^	69.6 ± 11.0 ^a^	4.9 ± 0.9 ^a^
C+Syk-	74 ± 8	76.9 ± 10.4 ^a^	67.3 ± 9.0 ^a^	4.8 ± 0.8 ^a^
C+LynI	74 ± 12	76.3 ± 9.4 ^a^	68.3 ± 9.5 ^a^	4.7 ± 0.5 ^a^
C++	72 ± 6	75.9 ± 12.7 ^a^	67.6± 9.8 ^a^	4.9 ± 0.7 ^a^

^a^: *p* < 0.001, comparing each parameter under different treatment against T_0_, by using Dunnett’s test, following a significant one-way ANOVA; no significant difference was observed comparing each treatment against C. Values are expressed as the mean ± SD.

## Supplementary Materials

Supplementary materials can be found at https://www.mdpi.com/1422-0067/21/11/4063/s1.

## Abbreviations

ARC	Acrosome Reacted Cells
NVC	Not viable Cells
CTB	Cholera Toxin beta subunit
SLC4A1	Solute channel 4 A1
