# Supplementary material for: Human Sperm Capacitation Involves the Regulation of the Tyr-Phosphorylation Level of the Anion Exchanger 1 (AE1)
Source: Int J Mol Sci. 2020 Jun 5;21(11):4063. doi: 10.3390/ijms21114063 (PMC7311965; doi:10.3390/ijms21114063)
Supplement: Supplementary file 1 [file ijms-21-04063-s001.pdf]

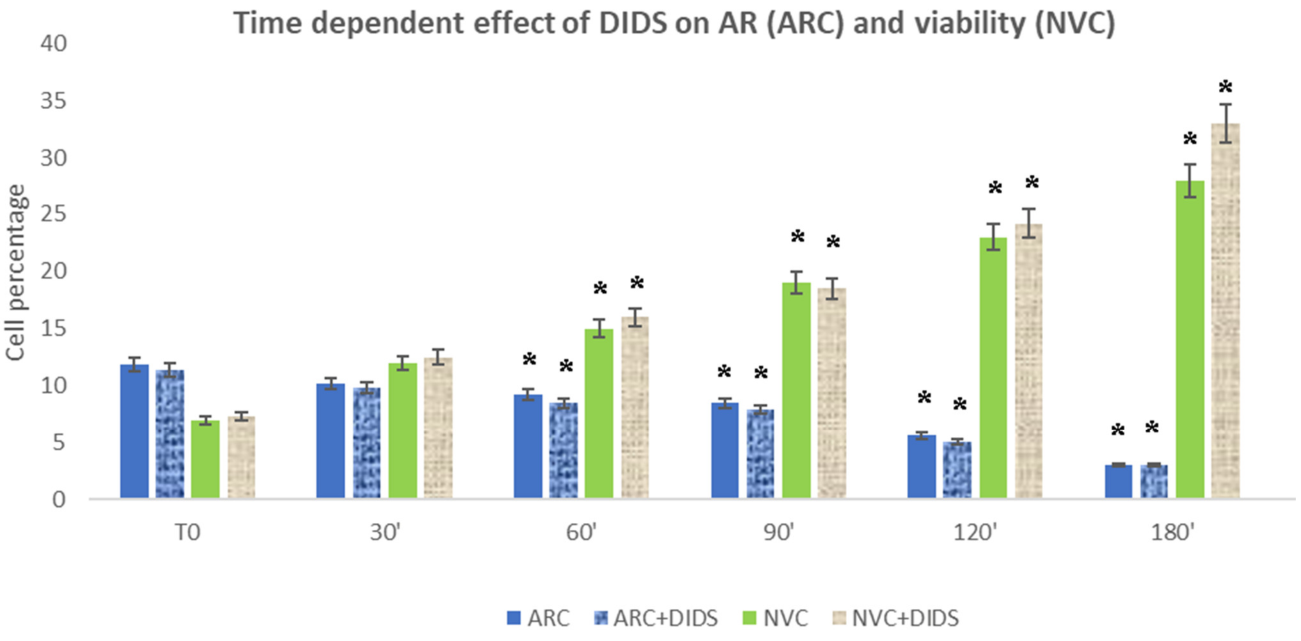

Figure S1. Time-dependent effect of 100 µM DIDS on sperm AR and viability. Sperm, isolated and purified as described in Methods, were resuspended in non-capacitating buffer (BWW in the absence of HCO_3-) and immediately processed for AR and viability (detected with PI labeling of not viable cells: NVC at (T0) or incubated for up to 180 min in the absence or presence of DIDS in PSW without BSA. Sperm were analyzed for acrosome-reacted cells (ARC) and viability, NVC, by immunofluorescence cytochemistry (see Methods). Values are expressed as percentages of cells. Comparison to T0 was performed by one-way analysis of variance (ANOVA) with Dunnett's *post hoc* test. Values represent the mean S.D. of at least 8 experiments. *Comparison to T0, $p<0.005$; **.

Table S1. Sperm motility and kinematic parameters observed in different samples. Motility and kinematic parameters of spermatozoon were evaluated with computer-assisted sperm analysis (CASA) at T_0 (before starting incubation) and after 180 min of incubation in capacitating conditions in absence (C) or presence of DIDS (1, 50 or 100 µM), I-172 (1, 5, 10 µM) or both (C+100 µM DIDS + 5 µM I-172). Motility = progressive and non-progressive motility (%); VSL = straight-line velocity (µm/s); VAP = average path velocity (µm/s); ALH = amplitude of lateral head displacement (µm).

	**Motility (%)**	**VSL (µm/s)**	**VAP (µm/s)**	**ALH (µm)**
**T_0**	**69 ± 3.2**	**58.4 ± 8.9**	**54.0 ± 6.7**	**3.1 ± 0.5**
**C**	**76 ± 7.3**	**77.8 ± 13.9**	**69.6 ± 11.0**	**4.9 ± 0.9**
**C+10 DIDS-**	**58 ± 8.5^a**	**56.9 ± 10.4 ^{a}**	**59.3 ± 7.0 ^{a}**	**4.1 ± 0.8 ^{a}**
**C+50 DIDS**	**45.7 ± 12.0^a**	**45.3 ± 9.4 ^{a}**	**48.3 ± 9.5^a**	**3.6 ± 0.8 ^{a}**
**C+100DIDS**	**32.3±9.5^a**	**38.5±8.5 ^{a}**	**40.1 ± 5.5 ^{a}**	**2.8 ± 0.3 ^{a}**
**C+1 I-172**	**45.9±9.2^a**	**59.3±6.7 ^{a}**	**52.3 ± 9.4 ^{a}**	**3.8 ± 0.8 ^{a}**
**C+5 I-172**	**36.6±9.0^a**	**29.9±7.5 ^{a}**	**44.9 ±6.5 ^{a}**	**2.6 ± 0.5 ^{a}**
**C+10 I-172**	**28.4±8.6^a**	**20.7±4.9 ^{a}**	**33.5 ± 4.5 ^{a}**	**2.5 ± 0.4 ^{a}**
**C++**	**15.2± 0.6^a**	**0.9 ± 0.4 ^{a}**	**20.5 ± 0.8 ^{a}**	**2.0 ± 0.2 ^{a}**

[a]: $p < 0.01$, **comparing each parameter under different treatment against C, by using Dunnett's test, following a significant one-way ANOVA. Values are expressed as the mean ± SD.**

Table S2

Sperm lysates, immediately processed (T0) or incubated for 120 min in the absence (C) or presence of +5µM I-172 (C+I-172), 100µM DIDS (C+DIDS), or both (C++). were immunoprecipitated with anti AE1 or anti-Syk antibodies and immunoblotted with anti-P-Tyr antibodies.

90 kDa bands corresponding to the phosphorylated proteins were densitometrically estimated, normalized to the 90 kDa protein corresponding to AE1, as revealed on the AE1-IP after stripping and re-probing with anti-AE1 antibody, or to the 72 kDa Syk band, as revealed on the Syk-IP after stripping and re-probing with anti-Syk antibody, and statistically analyzed. Data show the means ±SD of relative units (RU) of six separate experiments. Comparison to C values: *p<0.001, Comparison of C to T0 values:**p<0.05. Student's t test for paired data.

	**Tyr-P**	
	**Anti-AE1-IP**	**Anti-Syk-IP**
T_0	253 ± 15	185 ± 17
C	754 ± 45**	470 ± 18**
C+I-172	225 ± 12 *	168 ± 22 *
C+DIDS	204 ± 11 *	192 ± 18 *
C++	115 ± 34 *	175 ± 20 *

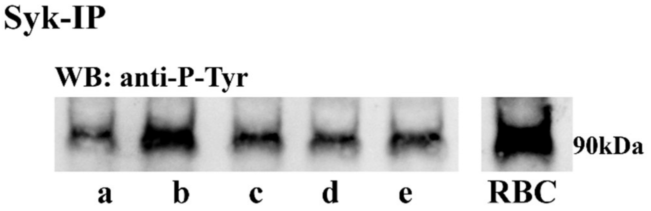

Figure S2. Co-immunoprecipitation of spAE1 with Syk. Sperm cells were either immediately processed (T0, lane a) or incubated for 120 min in the absence (C, lane b) or presence of +5µM I-172 (C+5I, lane c), 100µM DIDS C+DIDS, (lane d) or both (C++, lane e).). Sperm cells were then lysed and immunoprecipitated with anti Syk antibody and immunoblotted with anti-P-Tyr antibodies. The RBC lane shows the 90 kDa band corresponding to eAE1 revealed with anti-P-Tyr antibodies after probing a membrane lysate from human RBCs. The figure is representative of six separate experiments.

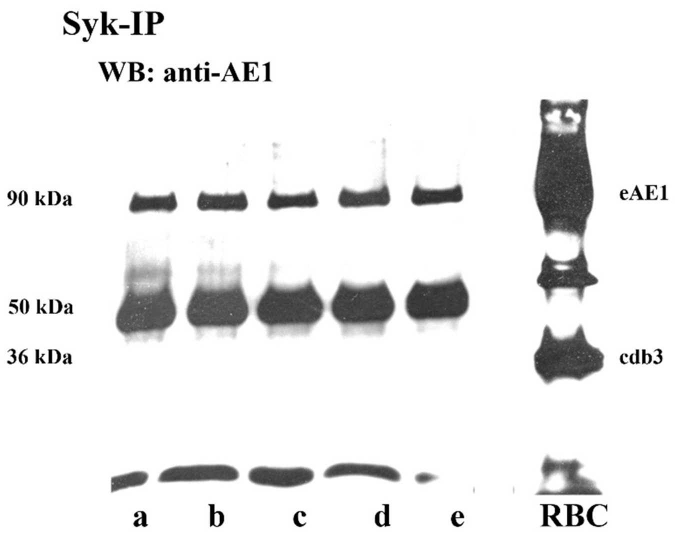

Figure S3. Sperm cells were either immediately processed (T0, lane a) or incubated for 120 min in the absence (C, lane b) or presence of +5◎M I-172 (C+5I, lane c), 100◎M DIDS C+DIDS, (lane d) or both (C++, lane e). Sperm cells were lysed and immunoprecipitated with anti Syk antibody and immunoblotted with anti-AE1 antibodies. The RBC lane shows the Western blotting profile revealed by probing erythrocyte membranes with anti-AE1 antibodies. The figure is representative of 6 separate experiments
